# Acupuncture for the treatment of tinnitus: a systematic review of randomized clinical trials

**DOI:** 10.1186/1472-6882-12-97

**Published:** 2012-07-17

**Authors:** Jong-In Kim, Jun-Yong Choi, Dong-Hyo Lee, Tae-Young Choi, Myeong Soo Lee, Edzard Ernst

**Affiliations:** 1 Department of Acupuncture and Moxibustion, College of Oriental Medicine, Kyung Hee University, Seoul, Republic of Korea; 2 School of Korean Medicine, Pusan National University, Yangsan, Republic of Korea; 3 Korean Medicine Hospital of Pusan National University, Yangsan, Republic of Korea; 4 Department of Korean Oriental Medical Ophthalmology & Otolaryngology & Dermatology, Wonkwang University Oriental Medical Center, Gunpo, Republic of Korea; 5 Present address: Office of Health Technology Evaluation, National Evidence-based Healthcare Collaborating Agency, Seoul, 110-450, Republic of Korea; 6 Medical Research Division, Korea Institute of Oriental Medicine, Daejeon, 305-811, South Korea; 7 Complementary Medicine, Peninsula Medical School, University of Exeter, Exeter, UK

**Keywords:** Acupuncture, Tinnitus, Systematic review, Alternative medicine, Effectiveness

## Abstract

**Background:**

Complementary and alternative medicine (CAM) has frequently been used to treat tinnitus, and acupuncture is a particularly popular option. The objective of this review was to assess the evidence concerning the effectiveness of acupuncture as a treatment for tinnitus.

**Methods:**

Fourteen databases were searched from the dates of their creation to July 4th, 2012. Randomized clinical trials (RCTs) were included if acupuncture was used as the sole treatment. The Cochrane risk of bias tool was used to assess the risk of bias.

**Results:**

A total of 9 RCTs met all the inclusion criteria. Their methodological quality was mostly poor. Five RCTs compared the effectiveness of acupuncture or electroacupuncture with sham acupuncture for treating tinnitus. The results failed to show statistically significant improvements. Two RCTs compared a short one-time scalp acupuncture treatment with the use of penetrating sham acupuncture at non-acupoints in achieving subjective symptom relief on a visual analog scale; these RCTs demonstrated significant positive effects with scalp acupuncture. Two RCTs compared acupuncture with conventional drug treatments. One of these RCTs demonstrated that acupuncture had statistically significant effects on the response rate in patients with nervous tinnitus, but the other RCT did not demonstrate significant effects in patients with senile tinnitus.

**Conclusions:**

The number, size and quality of the RCTs on the effectiveness of acupuncture for the treatment of tinnitus are not sufficient for drawing definitive conclusions. Further rigorous RCTs that overcome the many limitations of the current evidence are warranted.

## Background

Tinnitus is a common yet poorly understood disorder [1]. It is defined as the perception of sounds for which there is no external acoustic source [2]. Tinnitus is often associated with sudden, temporary hearing loss, and it can have a powerful detrimental impact on a patient’s quality of life. Tinnitus may result in sleep disturbances, work impairments and distress [3]. Approximately 10-15% of the general population experiences a persistent sensation of tinnitus. Tinnitus occurs most commonly among the elderly, but it can occur at any age. In many patients, the emergence of tinnitus occurs long after the disappearance of the underlying medical condition [2,4]. The pathophysiological mechanism of tinnitus is still unclear, and the disease is difficult to treat effectively [5]. There is firm clinical evidence that cognitive behavioral therapy, which is a type of psychotherapy, improves the quality of life of tinnitus patients [6]. However, no other therapies, including pharmacological treatment, can be considered to have well-established effectiveness in reducing tinnitus symptoms and the related quality of life [7-9].

Complementary and alternative medicine (CAM) has frequently been used to treat tinnitus, and acupuncture is a particularly popular option. Acupuncture is a therapeutic technique involving the insertion and manipulation of needles in the body. The use of acupuncture for treating the symptoms of tinnitus is similar to its use for pain relief because both conditions produce disagreeable sensory and emotional experiences [10]. The rationale for the use of acupuncture is the principle that a needle stimulus may elicit an electrical charge that triggers action potentials to rebalance the neurophysiological system or the function of the olivocochlear nucleus [11,12]. In the theory of traditional Asian medicine, tinnitus is closely connected to the yin-yang imbalance of the kidneys and of other internal organs such as the gallbladder because channels originating from these organs flow through the ear. Acupuncture is considered to be able to balance this skewed condition [13]. Several studies have reported that acupuncture can generate immediate relief from both the loudness and the disturbing quality of tinnitus, thereby resulting in a significant improvement in the quality of life [14,15]. Other studies have failed to show the effectiveness of the approach [16-18].

Dobie [7] summarized the clinical effectiveness of various alternative therapies, including acupuncture, in treating tinnitus. One systematic review published 12 years ago reported that the 6 randomized controlled trials (RCTs) cited did not produce convincingly positive effects [19]. However, these reviews did not perform systematic assessments [7] or are out of date [19].

The aim of this systematic review was to critically evaluate the current evidence from RCTs on the use of acupuncture as a symptomatic treatment for patients with tinnitus.

## Methods

### Data sources

The following databases were searched from their dates of creation through July 4th, 2012: MEDLINE, AMED, EMBASE, CINAHL, five Korean medical databases (Korean Studies Information, DBPIA, Korea Institute of Science and Technology Information, KoreaMed and the Research Information Service System), four Chinese medical databases (China National Knowledge Infrastructure databases: Chinese Academic Journals, the Century Journal Project, the China Doctoral/Master Dissertation full text database and the China Conference Proceedings full text database) and the Cochrane Library (2012, Issue 4). Two sets of search terms were used. The first set included terms for acupuncture, and the second set included terms for tinnitus (Additional file 1). The two sets were combined using the Boolean operator AND. The term ’acupuncture’ would also capture manual acupuncture, electroacupuncture (EA), scalp acupuncture and auricular acupuncture therapy. The Korean and Chinese terms for acupuncture and tinnitus were used in the Korean and Chinese databases. We also performed electronic searches of the relevant journals (Focus on Alternative and Complementary Therapies and Forschende Komplementarmedizin) through June 2012. Additionally, the reference lists of all the papers obtained were searched. Furthermore, our own personal files were manually searched. Hardcopies of all articles were obtained and read in full.

### Study selection

#### Types of studies

All prospective RCTs and quasi-RCTs were included in this review. Trials in which acupuncture was performed as part of a complex intervention were excluded. We excluded case studies, case series, qualitative studies, uncontrolled studies and controlled trials without randomization methods. Trials published in the form of dissertations and abstracts were included. No language restrictions were imposed.

#### Types of participants

Studies of patients of both sexes and any age with any type of tinnitus were included.

#### Types of interventions

Studies investigating the use of any type of needle acupuncture, with or without electrical stimulation, in tinnitus patients were included. We also included studies that examined the use of auricular acupuncture and scalp acupuncture in tinnitus patients. Trials with acupuncture as a concomitant treatment with other types of complementary therapies were excluded.

#### Types of controls

We included studies with control groups that received no treatment, sham acupuncture (i.e., penetrating insertion of a needle at a point other than a valid acupuncture point (a non-acupoint), superficial insertion at an acupuncture point or non-penetrating acupuncture at an acupuncture point or a non-acupoint) and relevant conventional drug therapies for tinnitus. Trials with designs that did not allow for the evaluation of the efficacy of the acupuncture (e.g., using other types of complementary therapies in the control group or comparing two forms of acupuncture or electroacupuncture) were excluded.

#### Types of outcome measures

Studies were required to include either the symptom severity or the relief of symptoms in tinnitus as outcome measures. Other clinically important outcomes included the quality of life, the response rate (i.e., responder vs. non-responder) and adverse events.

### Data extraction and quality assessment

Hard copies of all articles that appeared promising based on their abstract were obtained and read in full. All articles were read by three independent reviewers (JIK, MSL, and TYC), and data from the articles were validated and extracted according to pre-defined criteria (Table 1).

**Table 1:** Summary of randomized controlled trials of acupuncture for the treatment of tinnitus

**First author (year) [ref]**	**Sample size/ conditions (lesion)**	**Mean age or ranges (years) (Sex, M/F) Duration of disease (years)**	**Intervention (regimen)**	**Control (regimen)**	**Main outcomes**	**Main results (Effect size)**	**Design Country**
Hansen (1982) [16]	17 unilateral tinnitus	46 (9/8)	(A) AT (15 min, 2 times weekly for 3 weeks, n = 17)	(B) Sham AT (Penetrating, non-acupoints, 15 min, 2 times weekly for 3 weeks, n = 17)	Period index (ND with usual tinnitus + 2×ND with usual tinnitus + 3×ND with more pronounced tinnitus)	MD, 2.8 [-3.95, 9.55]	Cross-over
		5.3					Denmark
Vilholm (1998) [18]	53 severe tinnitus (both lesion)	53 (35/19)	(A) AT (30 min, total 25 treatment sessions over two months, n = 29)	(B) Sham AT (Penetrating, minimal, non-acupoints, 30 min, total 25 treatments over two months, n = 25)	1) Annoyance (VAS)	1) MD, -5.0 [-21.26, 11.26]	Parallel
		9.1			2) Loudness (VAS)	2) MD, -3.40 [-16.66, 9.86]	Denmark
					3) Awareness (VAS)	3) MD, -2.00 [-18.50, 14.50]	
Jeon (2012) [20]	33 unilateral tinnitus	A: 43.5 (13/4)	(A) AT (10min, 2 times weekly for 5 weeks, n = 17)	(B) Sham AT (Penetrating non acupoints, 10min, 2 times weekly for 5 weeks, n = 16)	1) Tinnitus Handicap Inventory score	1) Post, MD, 0.5 [-14.1, 15.1],NS	Parallel
		B: 49.6 (8/8)			2) Symptom I(VAS)	3mo, MD, -2.5 [-15.5,10.5], NS	Korea
		A: 37.4				2) Post, MD, -9.5 [-23.9, 4.9], NS	
		B: 45.6				3mo, MD, -12.5 [-27.0, 2.0], NS	
Wang (2010) [21]	50 tinnitus (n.r.)	30-70 (46/4)	(A) AT (25 min, once a week for 6 weeks, total 6 treatments, n = 19)	(C) Sham AT (Penetrating, minimal, non-acupoints, 25 min, once a week for 6 weeks, n = 15)	1) Subjective general evaluation of the treatments	1) A vs. C, MD, -0.02 [-0.12, 0.09], NS	Parallel
		n.r.	(B) EA (alternative frequency of 2/100 Hz at 3 s interval, 25 min, once a week for 6 weeks, total 6 treatments, n = 16)		2) Tinnitus occurrence	B vs. C, MD, 0.32 [0.24, 0.39], P < 0.0001	Denmark
					3) Tinnitus loudness	2) NS^†^	
					4) Quality reduction of daily life	3) NS^†^	
					5) Effect on the hearing improving	4) NS^†^	
						5) NS^†^	
Marks (1984) [17]	14 chronic unilateral tinnitus	51 (7/7)	(A) EA with (20 min, once weekly for two weeks, n = 14)	(B) Sham AT (Non-penetrating, non-acupoints, pricking and direct removal, once weekly for two weeks, n = 14)	1) Loudness at a particular time, loudness taking the day as a whole (VAS)	1) NS^†^	Cross-over
		n.r.			2) Response rate (verbal description of any changes)	2) NS^†^	UK
					3) Tinnitus matching score	3) NS^†^	
Okada (2006) [15]	76 tinnitus (n.r.)	57 (29/47)	(A) Scalp AT (manual rotation at 2 Hz, 15 seconds, one time, n = 38)	(B) Sham scalp AT (Penetrating, non-acupoints, manual rotation at 2 Hz, 15 seconds, one time, n = 38)	Subjective symptom relief (VAS)	MD, -1.34 [-2.48, -0.21], P = 0.02 in favor of A	Parallel
		n.r.					Brazil
de Azevedo (2007) [12]	38 tinnitus (n.r.)	36-76 (13/25)	(A) Scalp AT (manual rotation at 2 Hz, 15 seconds, one time, n = 19)	(B) Sham scalp AT (Penetrating, non-acupoints, manual rotation at 2 Hz, 15 seconds, one time, n = 19)	Otoacoustic emission amplitude	AT on the left side	Parallel
	Parallel	n.r.				- Right side: 3.39 [-1.53, 8.31], NS	Brazil
						- Left side: 1.35 [-3.29, 5.99], NS	
						AT on the right side	
						- Right side: 3.16[-0.99, 5.99], NS	
						- Left side: 1.56 [-2.21, 5.33], NS	
Tan (2007) [22]	90 nervous tinnitus (n.r.)	18-65 (48/42)	(A) AT (de-qi, 1 session = 20 min, daily,10 times, total 3 sessions, total 30 treatments, n = 30)	(B) Drug therapy (Bandazol, Dextran 40, Danshen tablet, and vitamin B12, 10 days, total 3 sessions, n = 30)	Response rate (verbal description of any changes)	RR, 2.20 [1.27, 3.89], P < 0.05	Parallel
		1 week-10years		*(C) Herbal medicine (n = 30)*			China
Jiang (2010) [23]	60 senile tinnitus (n.r.)	50-80 (21/39)	(A) AT (n.r., 1 session = 30 min, once daily, 5 times, rest for 2 days, 10 times/session, total 3 sessions, total 30 treatments, n = 30)	(B) Drug therapy (Flunarizine hydrochloride 10mg, orally at bedtime, 4weeks, stop taking without any improvements, effective service by adding 2 weeks, n = 30)	1) Hearing-threshold detection	1) MD, 0.28[-4.67,5.23], NS^‡^	Parallel
		n.r.			2) Activities of daily living (tinnitus, auditory, concomitant symptoms)	2) Tinnitus, MD, -0.75 [-1.10, -0.40], P < 0.0001; auditory, MD, -0.73 [-1.25, -0.21], P = 0.006; concomitant symptoms, MD, -0.27[-0.57, 0.03], NS	China
					3) Response rate (verbal description of any changes)	3) RR, 1.14 [0.87, 1.49], NS	

*MD*: mean difference; *ND*: Number of days; *n.r*.: not reported; *RR*: risk ratio; *VAS*: visual analogue scale; *NS*: not significant; ^†^No numerical data available for calculating effect size. We added the results on the base of authors’ results.

^‡^The original authors reported statistical significance but our calculation failed to do so.

The risk of bias was assessed using the Cochrane classification of seven criteria: random sequence generation, allocation concealment, patient blinding, assessor blinding, incomplete outcome data, selective outcome reporting and other risks of bias [24]. Because it is difficult to blind therapists to the use of acupuncture, we assessed patient and assessor blinding separately. We assigned low risk of bias for assessor blinding if the tinnitus was assessed by someone other than the therapist or the patient who did not know the group assignments. Disagreements were resolved by discussion between the three reviewers (JIK, MSL, and TYC).

## Results

### Study description

**Figure 1:**
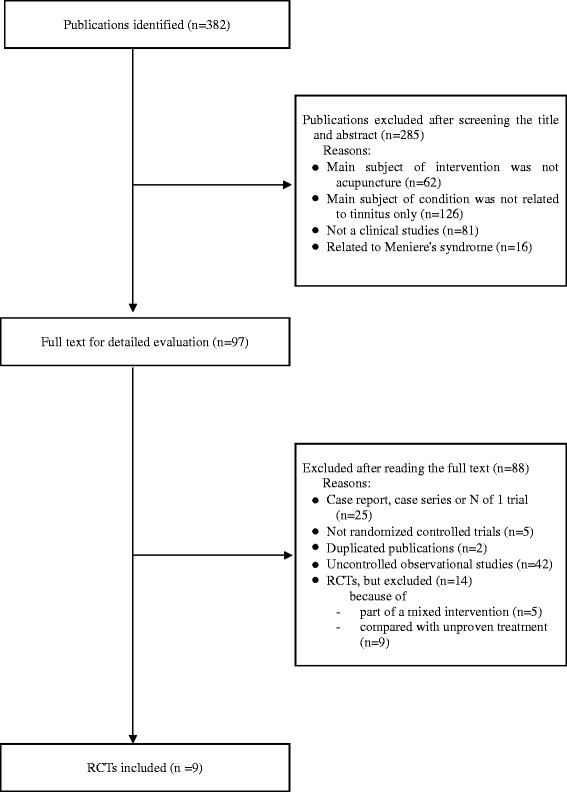
**Flowchart of the trial selection process.** RCT: randomized clinical trial; CCT: non-randomized controlled clinical trial; UCT: uncontrolled clinical trial.

The literature searches revealed 382 articles, of which 373 studies were excluded (Figure 1). Of these excluded studies, the reasons for the 14 excluded RCTs were as follows.

Five studies employed mixed interventions that included acupuncture in the active treatment group, including electroacupuncture plus herbal medicine injection [25], acupuncture plus gua-sha and cupping [26], acupuncture plus mixed administration of herbal and conventional medicines [27] and acupuncture plus herbal medicine ingestion [28,29]. Nine studies employed interventions of unproven efficacy in the control group, including another type of acupuncture [30-36], physiotherapy [37], and biofeedback in one control group and a third control medication in another group [38].

Nine RCTs met our inclusion criteria, and their key data are listed in Table 1[12,15-18,20-23]. Three of the included RCTs were from Denmark [16,18,21], two were from China [22,23], two were from Brazil [12,15], one was from England [17], and one was from Korea [20]. Five of the included trials adopted a two-armed parallel group design [12,15,18,20,23], two used a three-armed parallel group design [21,22], and two RCTs employed a crossover design [16,17]. Five trials employed manual acupuncture [16,18,20,22,23], one used electroacupuncture [17], one used manual acupuncture and EA [21], and two used scalp needle acupuncture [12,15]. The number of treatment sessions ranged from one to 30 sessions lasting from 15 seconds to 30 minutes each. The acupuncture points were selected according to traditional Chinese medicine (TCM) theory in eight RCTs [12,15-18,21-23] and protocol of previous study in one RCT [20] (Table 2).

**Table 2:** Summary of treatment points, their rationales and adverse events

**First author (Year)**	**De-qi manipulation**	**Total session**	**Acupuncture point**	**Rationales for selection of acupuncture points**	**Adverse events**
Hansen (1982) [16]	deqi sensation	6	Common: TE21,TE3,TE17, GB2, ipsilateral	TCM theory	n.r.
			Shi-type (hyper-function tinnitus): plus LR2		
			Xu-type (hypo-function tinnitus): plus KI3		
Vilholm (1998) [18]	deqi sensation	25	SI19, GB2,SI17, GV20: bilateral	TCM theory	n.r.
			Distal points, n.r.: individually, based on the		
			differentiation of signs and symptoms, bilateral.		
Jeon (2012) [20]	Deqi sensation	10	Prone: Bilateral local points: GB12, GB20	Previous study	n.r.
			Ipsilateral points: GV14,GV15,GV16,,GV20,GV21		
			Supine: Bilateral: BL2, LI20;		
			Ipsilateranl: TE12, TE22, SI19, GB2, TE17, GB7, GV20, EX-HN3		
Wang (2010) [21]	deqi sensation	6	Bilateral local points :GB8,TE17,GB2, GB20,GV20	TCM theory	n.r.
			Bilateral remote points :TE3,ST36		
Marks (1984) [17]	deqi sensation	2	LI4,LI5,SI4,SI5,SI19,KI6,PC9,GB11,GB12,TE17 and auricular point of vertigo	TCM theory	n.r.
Okada (2006) [15]	n.r.	1	Scalp acupuncture at cochleal-vestibular area (bilateral or ipsilateral n.r.)	TCM theory	Significant pain
					during needling, n = 2 (2.6%)
de Azevedo (2007) [12]	n.r.	1	Scalp acupuncture at cochleal-vestibular area (bilateral or ipsilateral n.r.)	TCM theory	n.r.
Tan (2007) [22]	deqi sensation	30	cervical Jiaji ( EX-B 2)	TCM theory	n.r.
Jiang (2010) [23]	n.r.	30	Point EX-HN1(Sishencong),TE21,GB2	TCM theory	n.r.
			Unilateral sick: ipsilateral acupoints		
			Bilateral sick: bilateral acupoints		

*TCM*: Traditional Chinese Medicine.

### Risk of bias

Four RCTs employed adequate methods of random sequence generation [12,15,20,23]. Two RCTs had a low risk of bias [20,23], but the risk of bias in the sequence generation was high in 2 RCTs [12,15]. One RCT employed allocation concealment [16]. Six of the 8 RCTs adopted both assessor and subject blinding [12,15-18,20], and another RCT adopted only assessor blinding [21]. Three RCTs had a low risk of bias due to incomplete outcome data criteria [16,20,21]. The risk of bias in selective outcome reporting was low in three RCTs [20-22] and high in one RCT [23] (Table 3).

**Table 3:** Risk of bias of included RCTs^*^

**Study**	**Random sequence generation**	**Allocation concealment**	**Patient blinding**	**Assessor blinding**	**Incomplete outcome data**	**Selective outcome reporting**
Hansen (1982) [16]	U	L	L	L	L	U
Vilholm (1998) [18]	U	U	L	L	U	U
Jeon (2012) [20]	L	U	L	L	L	L
Wang (2010) [21]	U	U	L	U	L	L
Marks (1984) [17]	U	U	L	L	U	U
Okada (2006) [15]	H	U	L	L	U	U
de Azevedo (2007) [12]	H	U	L	L	U	U
Tan (2007) [22]	U	U	U	U	U	L
Jiang (2010) [23]	L	U	U	U	U	H

^*^ Domains of quality assessment based on the Cochrane tools for assessing risk of bias.

Abbreviations; *L*: low risk of bias; *H*: high risk of bias; *U*: Unclear (uncertain risk of bias).

### Outcomes

#### Acupuncture vs. sham acupuncture

Seven RCTs tested the effectiveness of several types of acupuncture on symptoms of tinnitus comparing with penetrating or non-penetrating acupunctures [12,15-18,20,21].

Among them, five RCTs compared manual acupuncture or electroacupuncture on the symptoms of tinnitus to penetrating or non-penetrating acupuncture on acupoints [16-18,20,21]. The results failed to demonstrate statistically significant improvements in the periodic index, annoyance, loudness, awareness, Tinnitus Handicap Inventory, symptoms in visual analogue scale and response rate, although differences were found in some of subjective general evaluation of the treatment.

Two RCTs compared the effectiveness of a short one-time scalp acupuncture treatment to penetrating sham acupuncture at non-acupoints in achieving subjective symptom relief on a visual analog scale and in reducing the amplitude of otoacoustic emissions (OAE) [12,15]. One of these trials demonstrated significant effects of scalp acupuncture on symptom relief [12,15], but the other trial failed to significantly reduce OAE [12,15].

### Acupuncture vs. conventional drug therapy

Two RCTs compared acupuncture with conventional drug treatments, including Bandazol and Flunarizine hydrochloride [22,23]. One of these trials suggested that acupuncture had positive effects on the response rate in patients with nervous tinnitus [22], but the other trial failed to demonstrate improvement in patients with senile tinnitus [23].

## Discussion

Few sham-controlled RCTs have tested the effectiveness of acupuncture on tinnitus. Collectively, the results of the existing studies have failed to demonstrate specific effects of acupuncture. Only two of the 7 sham-controlled RCTs suggested positive effects. These two RCTs demonstrated beneficial effects of acupuncture for tinnitus when acupuncture was compared with drug therapy. However, the studies were too small to generate reliable findings. Both of the trials had small sample sizes [n < 40 for each group] and were thus prone to a type II error or an exaggeration of the treatment effect [39,40]. Overall, our findings provided no convincing evidence that acupuncture is beneficial for treating tinnitus.

We identified 6 new RCTs that had not been examined in previous reviews [12,15,20-23]. Nevertheless, the results of our review are similar to those of a previous review [19] that showed that acupuncture was not supported by sound evidence as a treatment for tinnitus. The previous review expressed concern regarding the poor methodological quality of the primary studies included.

Although most of the RCTs included in the present review employed sham-control groups, none was flawless. Five RCTs included fewer than 10 sessions [15-17,21,22], which may be insufficient for obtaining a positive clinical outcome. Two trials provided acupuncture in only a single session [15,22]. The likelihood of an inherent bias in the studies was assessed based on the description of the methods of randomization, blinding, and allocation concealment. Most of the included trials had a high risk of bias. Low-quality trials are more likely to overestimate the effect size [41,42]. None of the studies used a power calculation, and the sample sizes were usually small. Details regarding the dropouts and withdrawals were described in three trials [16,20,21]. However, the other RCTs did not report this information, which can lead to an exclusion or attrition bias. Consequently, the reliability of the evidence presented here is clearly limited.

Several sham acupuncture methods have been proposed for acupuncture trials. These methods include the penetrating insertion of needles at non-acupoints [12,15,16], superficially puncturing the skin at non-acupoints [18,20,21], and the non-penetration of non-acupoints [17]. In the present systematic review, no evidence for the superiority of real acupuncture over sham acupuncture was found, regardless of the acupuncture technique employed. Non-penetrating sham acupuncture has been reported to be superior to placebo tablets in reducing subjective pain [43]. This finding may suggest that most of the effects of needle acupuncture are nonspecific in nature.

Two RCTs from China compared acupuncture with conventional drug treatment and demonstrated positive effects on at least one outcome. However, the current evidence for the effectiveness of these drug therapies for treating tinnitus is not clear, and some drug therapies have been shown to be less effective than other forms of therapy. Additionally, the risk of bias in these RCTs was high, and the findings may be overestimated. Consequently, the evidence comparing acupuncture with drug therapies for the treatment of tinnitus is not clear.

Two RCTs compared the efficacy of scalp acupuncture with sham acupuncture for treating tinnitus[12,15]. Although the results demonstrated significant benefits from the scalp acupuncture, these trials also had a high risk of bias in randomization and allocation concealment. Consequently, these trials are likely to have overestimated the effect size.

The rationale for the acupuncture point selection was stated in all of the included RCTs. The authors in eight of the included RCTs quoted traditional Chinese theory as the justification for their point selection and used acupoints from the previous study in one RCT [20]. Needle stimulation resulting in the desired sensation (*de-qi*) in the patient is considered to be important for achieving maximal effects. This needle sensation was considered in the six RCTs included [16-18,20-22]. In the present data set, we found no evidence that the presence or absence of de-qi exerted an important influence on the clinical outcome.

Most of the included RCTs adopted questionnaires or self-reported inventories concerning the symptoms related to tinnitus. However, it is important to use only validated questionnaires. Unless the reliability and validity of the outcome measures used have been established, the data derived from the measures are subject to bias, and comparisons between the results of different studies are difficult.

In the included studies, several types of active acupuncture treatment were used: five RCTs used traditional manual body acupuncture [16,18,20,22,23]; one RCT used electroacupuncture [17]; one RCT used both manual body acupuncture and electroacupuncture [21]; and two RCTs used scalp acupuncture [12,15]. For all of these different types of needle stimulation, de-qi (i.e., sensation of needling in both practitioners and patients) might be a very important therapeutic factor in both traditional Asian medical viewpoint and neurophysiology [29]. However, the underlying neurophysiologic mechanism of these different acupuncture types according to the stimulating region (body versus scalp) and/or stimulating methods (manual versus electrical stimulation), might not be the same [44-46]. Therefore, the effect of different type of acupuncture on tinnitus should be interpreted apart and cannot be simply pooled altogether in future meta-analysis.

The experience and the number of the acupuncture practitioners used can influence the results of acupuncture clinical trials. Of the included trials, one trial [21] reported that one acupuncturist with more than 10 years of experience administered all of the treatments of real and sham acupuncture. No information was reported regarding the acupuncture practitioners in the other trials; consequently, we do not know whether the acupuncture was administered by well-trained practitioners.

Reports of adverse events from acupuncture are scarce, and those that have been reported are mild [47,48]. Adverse effects from acupuncture were reported in one of the reviewed RCTs [15]. Two cases of significant pain from the needles were reported. The fact that several RCTs did not clearly mention adverse effects demonstrates the poor reporting standards of acupuncture research.

Our review has a number of important limitations. Although substantial efforts were made to retrieve all RCTs on the subject, we cannot be certain that our search was exhaustive. Moreover, selective publishing and reporting are major causes of bias that should be considered [49-51]. It is conceivable that several negative RCTs remain unpublished, and this may distort the overall understanding of acupuncture. Further limitations include the paucity and the often-suboptimal methodological quality of the primary data. These factors influence both the quality and the quantity of the research reviewed. In total, these factors limit the conclusiveness of this systematic review.

## Conclusion

In conclusion, the evidence from sham-controlled RCTs testing the effectiveness of acupuncture for the treatment of tinnitus is not yet convincing. The number, size and quality of the RCTs are not sufficient for drawing definitive conclusions. Further rigorous RCTs that overcome the many limitations of the current evidence are warranted. However, in clinical practice, acupuncture treatment by experienced and licensed practitioners might be an option for tinnitus patients, especially when the patients refuse psychological behavioral therapy, which is the only treatment with clinical evidence demonstrating improved quality of life in tinnitus patients. Additionally, because acupuncture is a safe procedure and because there is no current treatment for which clinical efficacy has been demonstrated for specific tinnitus symptoms, acupuncture is an option for the treatment of tinnitus symptoms in patients who request this procedure in the clinic.

## Competing interest

The authors declare that they have no competing interest.

## Authors’ contributions

JIK, JYC, TYC and MSL designed the review, performed searches, appraised and selected trials, extracted data, contacted authors for additional data, carried out analyses and interpretations of the data, and drafted this report. DHL and EE reviewed and critiqued this review and report and assisted with interpretation of the data. All authors read and approved the final manuscript.

## Pre-publication history

The pre-publication history for this paper can be accessed here:

http://www.biomedcentral.com/1472-6882/12/97/prepub

## Supplementary Material

Additional file 1 Supplement 1. The search strategies. Click here for file
