# Supplementary material for: Acupuncture for the treatment of tinnitus: a systematic review of randomized clinical trials
Source: BMC Complement Altern Med. 2012 Jul 17;12:97. doi: 10.1186/1472-6882-12-97 (PMC3493359; doi:10.1186/1472-6882-12-97)
Supplement: Additional file 1 — Supplement 1. The search strategies. [file 1472-6882-12-97-S1.pdf]

Supplement 1. The search strategies

**MEDLINE (Ovid)**
1 exp tinnitus/<br>2 tinnit$.tw.<br>3 (ear$ and (buzz$ or ring$ or roar$ or click$ or plus$).tw.<br>4. or/1-3<br>5 acupuncture/<br>6 exp acupuncture therapy/<br>7 electroacupuncture/<br>8 meridians/<br>9 acupuncture points/<br>10 acupuncture$.tw.<br>11 (electroacupuncture or electro- acupuncture or acupoint$).tw.<br>12 (ear acupuncture or auricular acupuncture or scalp acupuncture or acupoint$).tw.<br>13 ((meridian or non-meridian) adj point$).tw.<br>14 or/5-13<br>15 4 and 14<br>16 limit 15 to human

**Cochrane (Wiley)**
1 exp tinnitus/<br>2 tinnit*:kw<br>3 (ear* and (buzz* or ring* or roar* or click* or plus*):kw<br>4 #1 and #3<br>5 MeSH descriptor Acupuncture<br>6 MeSH descriptor acupuncture therapy explode all trees<br>7 MeSH descriptor electroacupuncture<br>8 MeSH descriptor ear acupuncture<br>9 MeSH descriptor scalp acupuncture<br>10 MeSH descriptor meridians<br>11 acupuncture points/<br>12 acupuncture*:kw<br>13 (ear acupuncture or auricular acupuncture or scalp acupuncture or acupoint*):kw<br>14 ((meridian or non-meridian) NEAR point*):kw<br>15 #5 or #6 or #7 or #8 or #9 or #10 or #11 or #12 or #13 or #14<br>16 #4 and #15

Korean DBs
1 (이명 OR 귀울림) AND 침구 (ti., ab.)
2 (이명 OR 귀울림) AND 자침 (ti., ab.)
3 (이명 OR 귀울림) AND 경락 (ti., ab.)
4 (이명 OR 귀울림) AND 혈위 (ti., ab.)
5 (이명 OR 귀울림) AND 침자극 (ti., ab.)
6 (이명 OR 귀울림) AND 이침 (ti., ab.)
7 (이명 OR 귀울림) AND 두침 (ti., ab.)
8 (이명 OR 귀울림) AND 침 (ti., ab.)

CNKI
1 耳鸣 AND 针灸 (ti., ab.)
2 耳鸣 AND 针刺 (ti., ab.)
3 耳鸣 AND 经络 (ti., ab.)
4 耳鸣 AND 穴位 (ti., ab.)
5 耳鸣 AND 电针 (ti., ab.)
6 耳鸣 AND 耳针 (ti., ab.)
7 耳鸣 AND 头针 (ti., ab.)
